# A high working memory load prior to memory retrieval reduces craving in non-treatment seeking problem drinkers

**DOI:** 10.1007/s00213-017-4785-4

**Published:** 2017-11-27

**Authors:** Anne Marije Kaag, Anna E. Goudriaan, Taco J. De Vries, Tommy Pattij, Reinout W. Wiers

**Affiliations:** 1 Addiction Development and Psychopathology (ADAPT) Lab, Department of Developmental Psychology, University of Amsterdam, Amsterdam, The Netherlands; 2 Amsterdam Brain and Cognition, University of Amsterdam, Amsterdam, The Netherlands; 3 Department of Psychiatry, Academic Medical Center, University of Amsterdam, Amsterdam, The Netherlands; 4 Arkin Mental Health Care & Amsterdam Institute for Addiction Research, Amsterdam, The Netherlands; 5 Department of Anatomy and Neurosciences, Amsterdam Neuroscience, VU University Medical Center, Amsterdam, The Netherlands

**Keywords:** Memory reconsolidation, Craving, Alcohol, Working memory, Skin conductance

## Abstract

**Background:**

Reconsolidation-based interventions have been suggested to be a promising treatment strategy for substance use disorders. In this study, we aimed to investigate the effectiveness of a working memory intervention to interfere with the reconsolidation of alcohol-related memories in a sample of non-treatment seeking heavy drinkers.

**Methods:**

Participants were randomized to one of the two conditions that underwent a 3-day intervention: in the experimental condition, a 30-min working memory training was performed immediately after a 15-min memory retrieval session (i.e., within the memory reconsolidation time-window), whereas in the control condition, the working memory training was performed prior to a memory retrieval session.

**Results:**

In contrast to our original hypothesis, a high working memory load after memory retrieval did not interfere with the reconsolidation of those memories while a high working memory load prior to memory retrieval (the original control condition) strongly reduced retrieval-induced craving and craving for alcohol at follow-up.

**Conclusion:**

Whereas the neurocognitive mechanism behind this effect needs to be further investigated, the current findings suggest that, if replicated, working memory training prior to addiction-related memory retrieval has the potential to become an effective (adjunctive) intervention in the treatment of substance use disorders.

**Electronic supplementary material:**

The online version of this article (10.1007/s00213-017-4785-4) contains supplementary material, which is available to authorized users.

## Introduction

Substance use disorders (SUD) are characterized by the acquisition of maladaptive instrumental (drug-seeking and drug-taking) and Pavlovian (cue-drug associations) memories (Milton 2013). Disrupting reconsolidation of these memories has been proposed as a treatment strategy in SUD (Taylor and Torregrossa 2015; Torregrossa and Taylor 2015). When retrieved, consolidated memories enter an instable state, after which they are reconsolidated back into a stable long-term memory. By disrupting this reconsolidation process (Nader 2003; Torregrossa and Taylor 2015), it is hypothesized that substance-related memories become weaker and therefore have a less profound effect on addictive behavior, reducing substance-seeking and taking behavior(Milton and Everitt 2010).

Memory reconsolidation is a process that involves a sequence of molecular changes, including NDMA receptors, (nor)adrenergic signaling, glucocorticoid receptors, GABA, and several intracellular signaling molecules (Tronson and Taylor 2013; Taylor and Torregrossa 2015). Most preclinical studies used pharmacological agents to disrupt reconsolidation of addiction-related memories, including the beta-adrenergic receptor antagonist propranolol. When administered following memory reactivation, propranolol reduces drug-seeking behavior in animals (Bernardi et al. 2006; Milton et al. 2008), whereas in humans, it impairs reconsolidation of drug-related words (Zhao et al. 2011) and it reduces craving in substance-dependent patients (Saladin et al. 2013; Lonergan et al. 2016).

While several pharmacological agents have proven to be highly effective in animals, the translation of these effects to humans is problematic due to a variety of reasons. For example, these pharmacological agents may not disrupt all of the Pavlovian representations influencing instrumental substance-seeking behavior (Milton and Everitt 2010). Moreover, many pharmacological agents that block memory reconsolidation are highly toxic and unsuitable for human use (Das et al. 2015a; Beckers and Kindt 2017). Most importantly, a precise timing between the administration of a pharmacological agent and memory retrieval is crucial for a successful blockage of memory reconsolidation (Barbara 2012; Elsey and Kindt 2017). Therefore, behavioral interventions targeting reconsolidation of substance-related memories may be better suited for the treatment of SUD, because behavioral interventions are not hampered with these limitations associated with pharmacological blockage of reconsolidation.

In this study, we investigate the effectiveness of a novel memory retrieval (MR)-working memory (WM) protocol to disrupt the reconsolidation of substance-related memories. In most clinical studies, WM training is used as an adjunctive intervention for the treatment of SUD aimed at increasing executive functioning (Bickel et al. 2014). These interventions have small to moderate effects on reducing substance use (Houben et al. 2011; Rass et al. 2015; Verdejo-Garcia 2016) and impulsive behavior in stimulant drug dependence(Bickel et al. 2012). In addition, several studies have demonstrated that a high WM load during retrieval of substance-related memories reduces craving for cigarettes (May et al. 2010) and food (Andrade et al. 2012; Kemps and Tiggemann 2013; McClelland et al. 2006; Steel et al. 2006). A possible explanation for these effects is that a high WM load following memory retrieval interferes with the reconsolidation of those memories as it has recently been demonstrated that a WM task after the retrieval of trauma-related memories (thus during memory reconsolidation) reduces intrusion of these memories (James et al. 2015). This effect has been attributed to competition between WM resources that are both needed for successful WM performance as well as memory (re)consolidation, including, but not limited to, overlapping regions in the medial prefrontal cortex (Bechara et al. 1998; Laroche et al. 2000; Barsegyan et al. 2010; Sierra et al. 2017). However, it remains to be investigated whether a WM task also interferes with reconsolidation of addiction-related memories.

In the current study, 57 non-treatment seeking problem drinkers were included and randomized to a MR-WM condition or a WM-MR condition. All participants underwent three intervention sessions, preceded by a baseline assessment session and a post-intervention assessment session. In the MR-WM condition, a 30-min WM training took place following a short memory retrieval session, whereas in the WM-MR condition, WM training took place before the memory retrieval session. It was hypothesized that a high WM load following memory retrieval (that is, within the reconsolidation time-window) would disrupt the reconsolidation of these memories, whereas a high WM load preceding memory retrieval (that is, outside the reconsolidation time-window) would not disrupt the reconsolidation of these memories.

Since modulation of reconsolidation is expected to alter alcohol-related memories, the primary outcome measures were conditioned responses (e.g., craving and physiological reactivity) during memory retrieval on the day before and the day after the 3-day intervention. It was expected that participants in the MR-WM condition would show greater reductions in retrieval-induced craving and physiological reactivity, and a greater reduction in alcohol intake and craving at follow-up, compared to participants in the WM-MR condition. In addition, participants in the MR-WM conditions, compared to participants in the WM-MR condition, were expected to show greater reductions in desire for alcohol and alcohol intake at 1-week follow-up and 1-month follow-up.

## Methods

### Participants

Fifty-seven heavy drinkers (27 females) were included in the study. Participants were recruited through internet and poster advertisement in the local community of Amsterdam and the Psychology faculty of the University of Amsterdam. After providing informed consent, participants received an online screening questionnaire, to assess age, alcoholic beverage of preference, and drug use in the last 12 months. Participants were asked to indicate whether they wanted to stop drinking, whether they wanted to reduce their drinking, or whether they did *not* want to change their drinking. In addition, alcohol use severity was assessed using the Alcohol Use Disorder Identification Test (AUDIT; Saunders et al. 1993). Participants who indicated to have used a certain drug more than 40 times in the past 12 months were asked to fill-out the Drug Use Disorder Identification Test (DUDIT; Berman et al. 2003, 2005) to assess drug use severity. Inclusion criteria were an age between 18 and 40, a total AUDIT score of 12 or higher, and a preference for beer or wine. Participants were excluded if they had a DUDIT score of 12 or higher or when they were not motivated to change their drinking. Participants received a monetary compensation or research participation credits upon completion of the study. The study was approved by the psychology ethics committee of the University of Amsterdam.

### Design

Participants were pseudo-randomly assigned to the experimental or control condition, stratified by gender, age (18–29 or 29–40), and AUDIT scores (12–19, 20–26, 27–33, 34–40) and scheduled for five consecutive sessions. All participants were blind to the experimental condition and were informed that the aim of the study was to investigate the effectiveness of WM training and alcohol memory activation on alcohol intake.

**Fig. 1:**
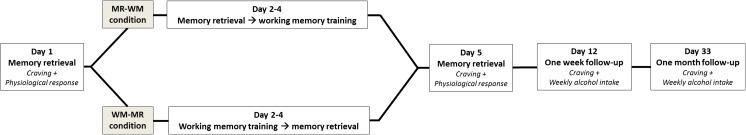
The experimental design. Participants were randomized to the experimental condition (MR-WM) or the control condition (MR-WM). In the experimental condition, they first did a memory retrieval session that was followed by a working memory task, whereas in the control condition, they first performed a working memory task that was followed by a memory retrieval session

All study procedures took place between 15.00 and 23.00 h to minimize the effects of diurnal variations in craving (West, Schneiders 1987). In session 1 and session 5, pre- and post-intervention measures of retrieval-induced desire for alcohol and changes in skin conductance levels (SCL) and heart rate were taken as described below. In sessions 2, 3, and 4, the MR-WM training or WM-MR training was performed (see Fig. 1). Participants were instructed not to consume any alcohol during the 5 days of the experiment. Compliance to this instruction was assessed using the Timeline Followback (TLFB; Sobell and Sobell 1992) at session 5 and a breathalyzer test for alcohol at the start of each session. Participants with a positive breathalyzer test or participants that indicated to have drank something after one of the intervention days were excluded from analyses.

#### Session 1: baseline measure of cue reactivity and mental state

Participants filled-out an online questionnaire to assess alcohol intake in the 2 weeks preceding the first session, using the TLFB. The Desire for Alcohol Questionnaire (DAQ; Love et al. 1998) was used to assess craving on three subscales: craving related to the desire for alcohol, craving related to the negative reinforcement of alcohol, and craving related to the loss of control over drinking. The stage of motivation to change drinking patterns (e.g., pre-contemplation, contemplation, or action stage) was assessed using the Readiness to Change Questionnaire (RCQ; Heather et al. 1991). After filling out the questionnaires, all participants underwent a 30-min MRI scan and an approach-avoidance task, of which the results will be presented elsewhere.

For the WM task and memory retrieval session, participants were taken into a sound-attenuated room. After connecting the electrodes for the ECG and SCL measures, the experimenter left the room and the memory retrieval session started. After completing the memory retrieval session, participants went home after being instructed not to consume any alcohol during the following 4 days.

#### Sessions 2–4: MR-WM training or WM-MR training

In sessions 2–4, all participants underwent the MR-WM intervention or the WM-MR intervention. Similar to session 1, these sessions started with the placement of the ECG and SCL electrodes, after which the experimenter left the room and the intervention started. After completion of the retrieval session, the experimenter came in to either detach the electrodes (for participants in the WM-MR condition) or to take away the alcohol and to start the WM task (for the participants in the MR-WM condition). The 30-min WM task and the 6-min memory retrieval session took place immediately after each other, and the whole session took approximately 45 min.

#### Session 5: post-intervention measure of cue reactivity and mental state

The experimental procedures in session 5 were similar to those in session 1, with the exception of the RCQ that was not assessed. The TLFB was included in this session to assess self-reported alcohol intake during the week that participants were enrolled in the experiment.

#### Take-home questionnaires and follow-up at 1 week and at 1 month

After session 1, participants received an e-mail with an online questionnaire to assess demographic information, smoking severity using the Fagerstrom Test for Nicotine Dependence (FTND; Heatherton et al. 1991), cannabis use severity (CUDIT; Adamson and Sellman 2003), and psychological symptoms and psychological distress using the Symptom Checklist 90 (SCL90; Derogatis and Unger 2010). At 1 and 4 weeks of follow-up, participants received an online questionnaire to DAQ, alcohol intake in last 7 days (TLFB), and drug use in the last 7 days.

### Memory retrieval protocol

**Fig. 2:**
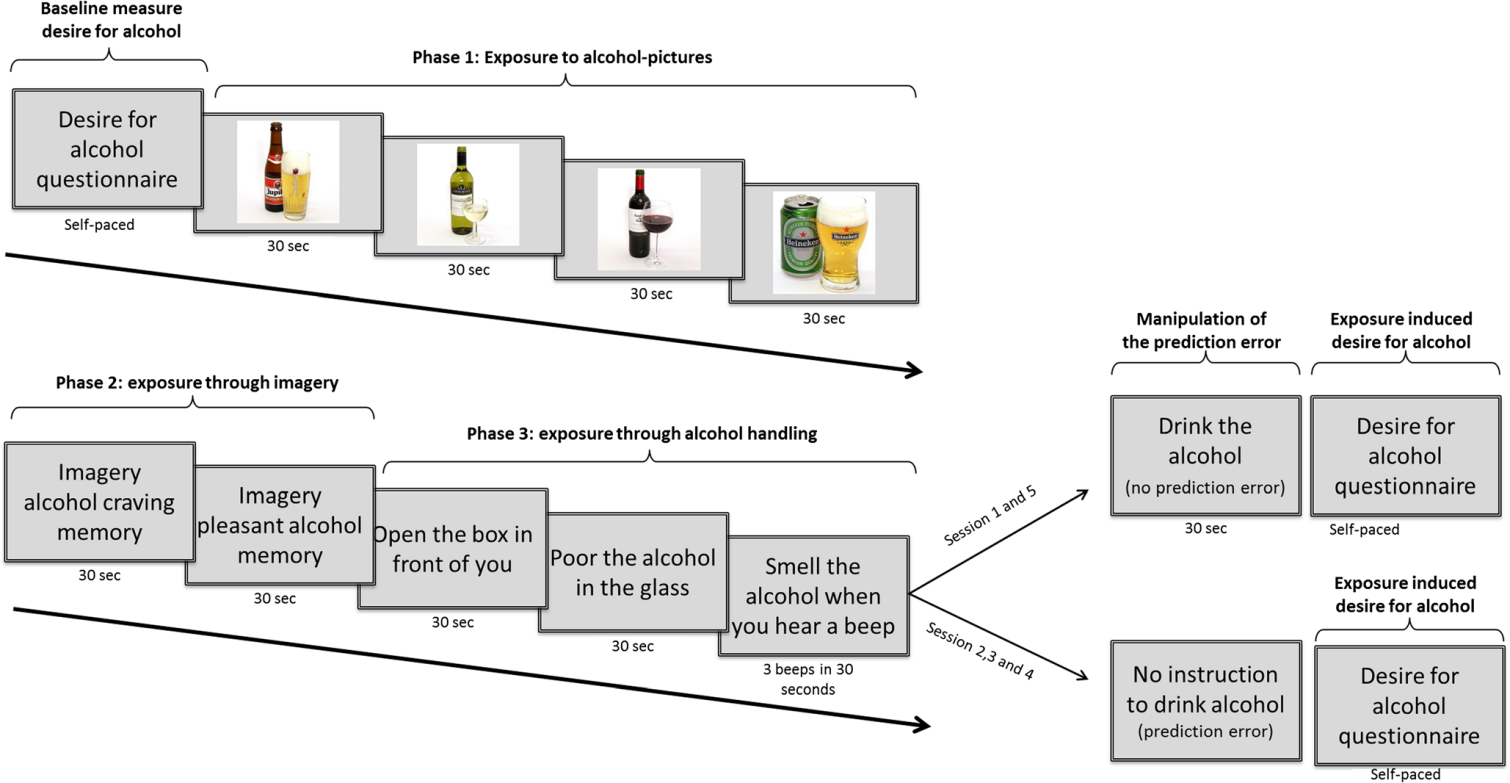
The memory retrieval session was fully computer-assisted and consisted of the baseline assessment of craving, the presentation of four alcohol pictures, the reading of two personalized scenarios of alcohol-related memories, and the handing of alcohol (holding, pouring, smelling). In sessions 1 and 5, the last instruction was to take a sip of the alcohol, whereas this instruction was not given in sessions 2, 3, and 4 to induce a prediction error

The computer-assisted alcohol memory retrieval paradigm was modified from a protocol by Hammarberg et al. (2009) and Khemiri et al. (2015) (Fig. 2). The retrieval session started with the baseline assessment of craving using the DAQ, followed by a short relaxation exercise. Next, the memory retrieval started with the presentation of four alcohol-related pictures that were all shown for 30 s. Subsequently, participants were instructed to read out loud two personalized scenarios of alcohol-related situations. These 30-s scenarios were based on information provided by the participants prior to the start of the experiment and described a pleasant alcohol-related memory and a memory of a situation that induced strong feelings of craving. Thereafter, the in vivo exposure phase started in which participants were instructed to open a box that was in front of them, to take out the alcohol, to poor the alcohol into a glass, and to smell the alcohol three times. Participants had 30 s to perform each instruction. At the end of the memory retrieval sessions, craving was assessed again using the DAQ. More details of the task are described in the supplementary methods.

Because a prediction error is suggested to be critical to destabilize memories and to induce memory reconsolidation (Fernández et al. 2016; Sevenster et al. 2014; Taylor and Torregrossa 2015), the memory retrieval protocol in sessions 2, 3, and 4 did not include the last instruction to drink the alcohol (Das et al. 2015b). Compliance to all instructions was confirmed by observing the participants.

### WM task

**Fig. 3:**
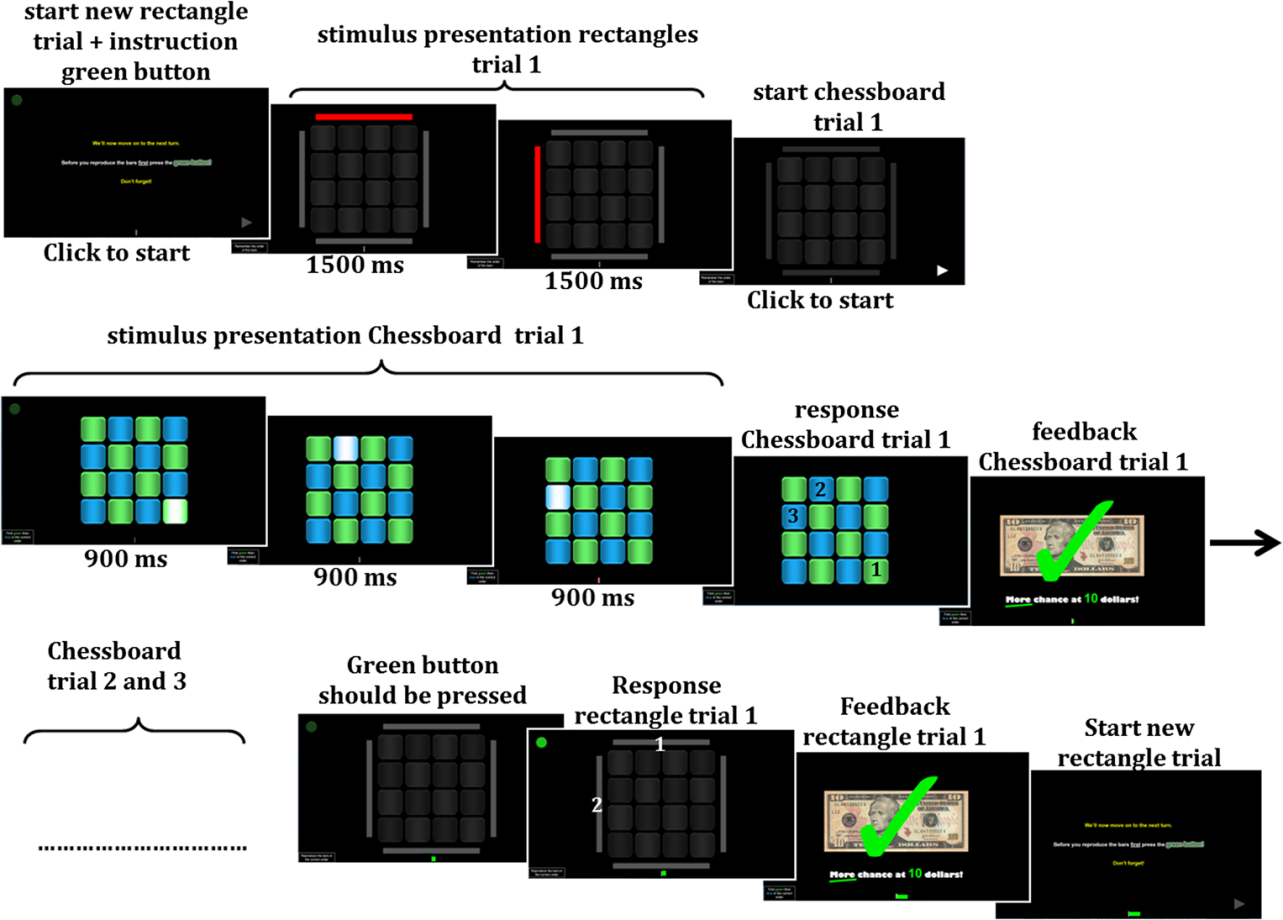
The complex chessboard task. The visuospatial working memory task consisted of 19 blocks of one rectangle trial and three chessboard trials. The task took approximately 30 min and task level was individually adjusted

The complex chessboard task (Fig. 3), developed by Dovis et al. (2012), is a visuospatial WM task based on the Corsi block tapping task (Corsi 1973) and the subtest Letter-Number Sequencing from the Wechsler Adult Intelligence Scale (WAIS; Wechsler 1958). In short, participants performed a total of 76 trials that took approximately 30 min. Task difficulty was individually adapted so that all participants successfully completed the task that was rewarded with €10. The complex chessboard task assesses the ability to both maintain (remember) and manipulate a sequence of blue and green squares that light up in a random order and have to be repeated in a specific order (first the green squares, then the blue squares). WM load in this task is measured by the maximum length of the chessboard sequence that is successfully remembered and manipulated. More details of the task are described in the supplementary methods.

### SCL and heart rate

SCL and heart rate were measured continuously during all retrieval sessions. Vsrrp98 software was used to record and analyze the data (Vsrrp98 v10.4, University of Amsterdam, 1998–2017). More details are described in the supplementary methods.

### Statistical analyses

One way ANOVAs (or non-parametric equivalents in case of violation of normality) were used to test for baseline group differences in age, alcohol use severity, drug use severity, smoking severity, weekly alcohol intake, and alcohol craving. Chi-square tests were used to test whether the experimental groups significantly differed in the readiness to change stage and drug use (cocaine, amphetamine, speed, heroine, XTC, LSD, ketamine, cannabis) in the past 12 months that was measured on an ordinal scale (no drug use, 1–2 times, 3–5 times, 6–10 times, 11–20 times, 21–40 times, or more than 40 times).

To test for the effect of intervention condition on retrieval-induced craving, repeated measures analyses were performed on the total DAQ scores with condition as between group factor and session (session 1 versus session 5) and retrieval phase (pre- or post-retrieval) as within group repeated measures factor. Only significant results on total DAQ scores were followed-up by three separate repeated measurements ANOVAs on the three different subscales to assess whether differences in craving were specifically related to craving for alcohol-related to desire, craving for alcohol-related to negative reinforcement, or craving for alcohol-related to loss of control.

Mean SCL and heart rate were calculated for each retrieval phase (craving before retrieval, relaxation, exposure to alcohol pictures, alcohol memory imagery, alcohol handing, and craving after retrieval). The effect of intervention condition on SCL and heart rate was tested using a repeated measures ANOVA with intervention condition as between group factor, with session (session 1 and session 5) and the six retrieval phases as within group repeated measures factor. Planned polynomial contrast was used to assess the curve characteristics.

The same analyses were done for the data (retrieval-induced desire for alcohol, SCL, and heart rate) acquired during the intervention sessions, with sessions 2, 3, and 4 (instead of session 1 and 5) as repeated measures factor.

To test for the long-term effects of the intervention on overall craving and alcohol consumption, repeated measures analyses were performed on the total DAQ scores (and in case of a significant effect also on the three subscales of the DAQ) and weekly alcohol intake with intervention condition as between group factor and time (baseline, 1-week follow-up, and 1-month follow-up) as within group repeated measures factor. Simple planned comparisons were used to test for changes compared to baseline.

In order to analyze WM capacity, the first 12 trials (nine chessboard trials and three rectangle trials) were excluded from analyses because participants typically need a certain number of trials to reach an optimal difficulty level (Dovis et al. 2012). The remaining 64 were divided into four blocks of 12 chessboard trials and 4 rectangle trials. For these four blocks, the mean length of the chessboard sequence was calculated, as well as the number of errors in the chessboard trials and rectangle trials. Three repeated measures analyses were used to test for the main and interaction effects of treatment condition and session, in chessboard sequence length, chessboard errors, and rectangle errors.

The mean sequence length over the three training sessions was used to explore whether participants that performed better on the WM task (those who had a higher WM load) showed stronger changes in retrieval-induced response and stronger changes in craving at 1-week and 1-month follow-up. We therefore categorized all participants on low or high WM (based on the median-split of the mean sequence lengths during all three intervention sessions) and included this as an extra factor in the exploratory analyses.

## Results

### Group characteristics

Of all participants, two reported to have consumed alcohol after one of the intervention sessions. Because alcohol consumption within the reconsolidation window may counteract the intervention effects on memory reconsolidation, these participants were excluded from all further analyses. As a result, a total of 28 participants (14 females) were included in the MR-WM condition and 27 participants (13 females) were included in the WM-MR condition. At baseline, groups did not significantly differ in age, AUDIT scores, readiness to change, total DAQ scores, smoking severity, weekly alcohol intake, and most of the drugs used. However, MDMA use (*χ* ^2^ = 7.90, *p* = 0.048) and cannabis use (*χ* ^2^ = 14.70, *p* = 0.023) use was slightly higher in the WM-MR group (see Table 1). Moreover, cannabis use severity was higher in the WM-MR group compared to the MR-WM group (*U* = 251, *p* = 0.019).

**Table 1:** Demographic and clinical information at baseline

	MR-WM group (*n* = 28)	WM-MR group (*n* = 27)	*p* value
age	22 ± 10	23 ± 7	*p* = 0.97
Sex (no. female)	14	13	*p* = 0.91
DAQ (craving)	2.92 ± 0.79 (S.D)	3.33 ± 1.11	*p* = 0.12
Desire for alcohol	3.12 ± 0.99 (S.D)	2.9 ± 0.97 (S.D)	
Negative reinforcement	3.43 ± 1.21 (S.D)	3.69 ± 1.41 (S.D)	
(Loss in) control	2.48 ± 1.13 (S.D)	3.5 ± 3.00 (S.D.)	
RCQ (readiness to change)			*p* = 0.61
Pre-contemplation phase	4% (n = 1)		
Contemplation phase	46% (*n* = 13)	44% (*n* = 12)	
Action phase	50% (*n* = 14)	56% (*n* = 15)	
Substance use^a^			
Weekly alcohol intake^b^	20 ± 13.13 (IQR)	23.5 ± 13 (IQR)	*p* = 0.50
MDMA use in past 12 months			
1–10 times	36% (*n* = 10)	41% (*n* = 11)	***p*** **= 0.048**
Cannabis use			***p*** **= 0.023**
1–20 times	39% (*n* = 11)	44% (*n* = 12)	
21–40 times	18% (*n* = 5)	33% (*n* = 9)	
> 40 times	14% (*n* = 4)	11% (*n* = 3)	
AUDIT (alcohol use severity)	17 ± 6.75 (IQR)	21 ± 7 (IQR)	*p* = 0.11
CUDIT (cannabis use severity)	0 ± 3 (IQR)	5 ± 9 (IQR)	***p*** **= 0.019**
FTND (smoking severity)	22 ± 10.75 (IQR)	18 ± 19 (IQR)	*p* = 0.06

Significance level is p<0.05

^a^Only drug use that differed between groups is shown in this table

^b^Number of standard units p<0.05

### The effects of a 3-day WM-MR intervention on retrieval-induced changes in desire for alcohol

**Fig. 4:**
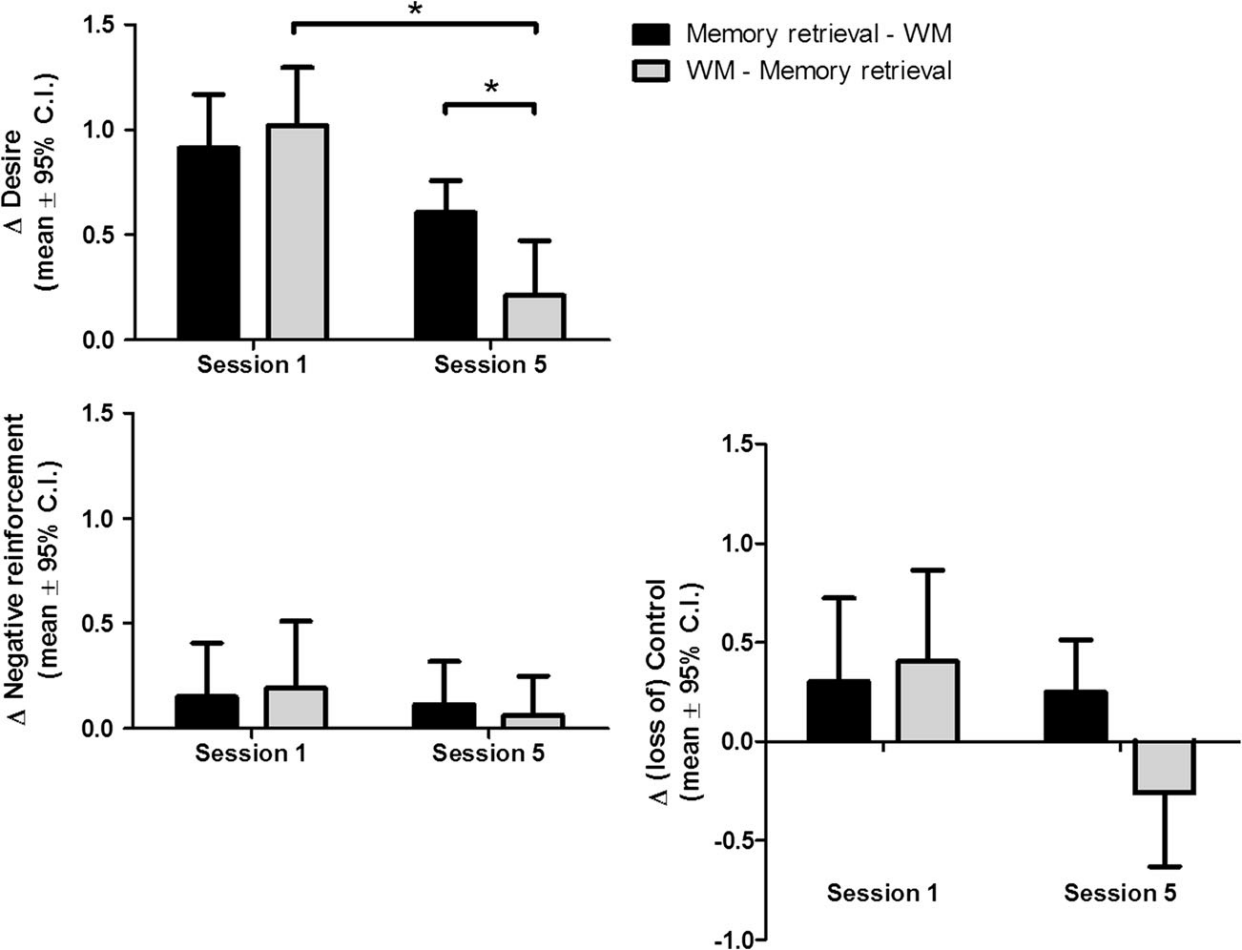
There was a retrieval by time by condition interaction effect only for DAQ-desire. Only participants performing a WM task prior to memory retrieval displayed a significant reduction in retrieval-induced desire for alcohol

Repeated measures ANOVAs were performed on the total DAQ scores as well as the DAQ subscales, with retrieval (pre- versus post-retrieval) and session (session 1 versus session 5) as repeated measures and condition as between subject factor. There was a significant exposure by session by condition interaction effect on total DAQ scores (*F*_1, 53_ = 4.89, *p* = 0.03, *η*_p_^2^ = 0.08). Repeated measures ANOVAs for each separate subscale demonstrated that this effect was specific for craving related to desire for alcohol (DAQ-desire: *F*_1, 53_ = 5.75, *p* = 0.02, *η*_p_^2^ = 0.10) but not craving related to negative reinforcement (DAQ-reinforcement) or craving related to loss of control (DAQ-control (Fig. 4). Follow-up analyses demonstrated that at baseline there were no differences between conditions in retrieval-induced desire for alcohol, whereas there were differences at session 5 (*F*_1, 53_ = 7.29, *p* = 0.009, *η*_p_^2^ = 0.12). In particular, participants in the MR-WM condition did demonstrate a significant retrieval-induced increase in DAQ-desire (*F*_1, 27_ = 66.04, *p* < 0.001, *η*_p_^2^ = 0.71), whereas participants in the WM-MR condition did not.

In contrast to our hypothesis, these results suggest that a high WM load prior to memory retrieval (WM-MR) reduces cue-induced craving, whereas a high WM load after memory retrieval, and thus during the reconsolidation window (MR-WM), had no effect on cue-induced desire for alcohol.

### The effects of a 3-day WM-MR intervention on skin conductance levels and heart rate during alcohol-cue exposure

Due to technical errors, data of 12 participants was incomplete (three males in MR-WM group, three females in MR-WM group, two males in the WM-MR group, four females in the WM-MR group). Therefore, the following analyses are done on a sample of *n* = 43.

**Fig. 5:**
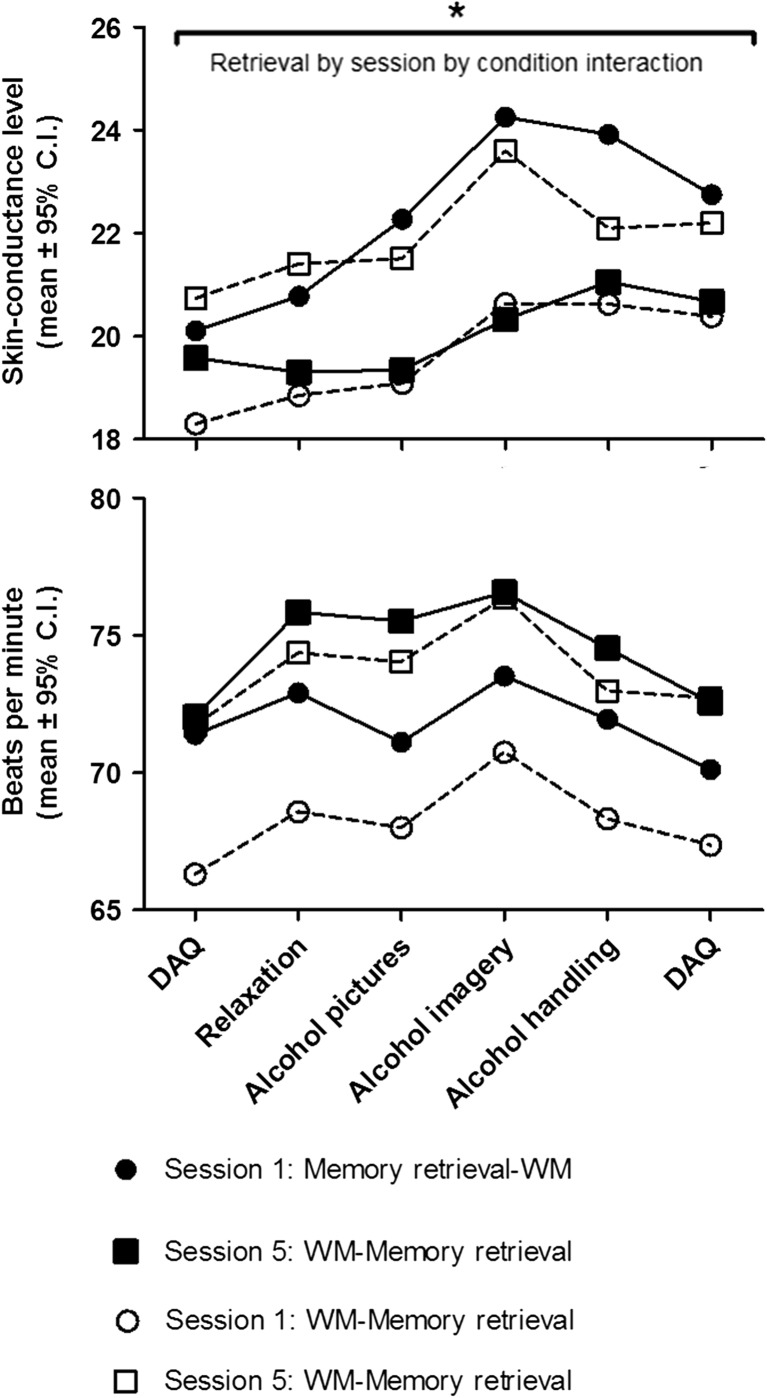
There was a significant retrieval by session by condition quadratic interaction effect for skin conductance levels. Only for participants in the memory retrieval–WM condition skin conductance levels during retrieval significantly interacted with session

Two repeated measures analyses were performed with retrieval-phase and session as within group factor and intervention condition as between group factor. For SCL, there was a main effect of retrieval phase (*F*_5, 195_ = 22.13, *p* < 0.001, *η*_p_^2^ = 0.36) without other significant main and interaction effects. Planned polynomial contrasts, to test for differences in trends, showed that there was a significant cubic effect of retrieval phase (*F*_1, 39_ = 12.67, *η*_p_^2^ = 0.25) and a significant retrieval by session by condition quadratic interaction effect (*F*_1, 39_ = 22.86, *p* = 0.034, *η*_p_^2^ = 0.11) (Fig. 5). Within group tests revealed that there was a quadratic session by retrieval interaction effect in the MR-WM-only (*F*_1, 21_ = 10.85, *p* = 0.003, *η*_p_^2^ = 0.34). There was no session by retrieval interaction effect in the WM-MR condition. Altogether, these results suggest that SCL responsiveness during memory retrieval is more affected in the MR-WM condition compared to the WM-MR condition.

For heart rate, there was a main effect of retrieval phase (*F*_5, 35_ = 9.54, *p* < 0.001, *η*_p_^2^ = 0.58) and a main effect of session (*F*_1, 39_ = 8.38, *p* = 0.006, *η*_p_^2^ = 0.18). Planned polynomial contrasts showed that there was a quadratic effect of retrieval on heart rate (*F*_1, 39_ = 44.00, *p* < 0.001, *η*_p_^2^ = 0.53) (Fig. 5).

### Intervention effects on self-reported desire for alcohol and weekly alcohol intake at 1-week and at 1-month follow-up

To investigate the long-term effects of the intervention, we performed repeated measures analyses on the DAQ scores at the beginning of session 1, at 1-week follow-up, and at 1-month follow-up. There was a significant main effect of time (*F*_2, 104_ = 24.75, *p* < 0.001, *η*_p_^2^ = 0.32) and a significant condition by time interaction effect on total DAQ scores (*F*_2, 104_ = 0.16, *η*_p_^2^ = 0.08).

**Fig. 6:**
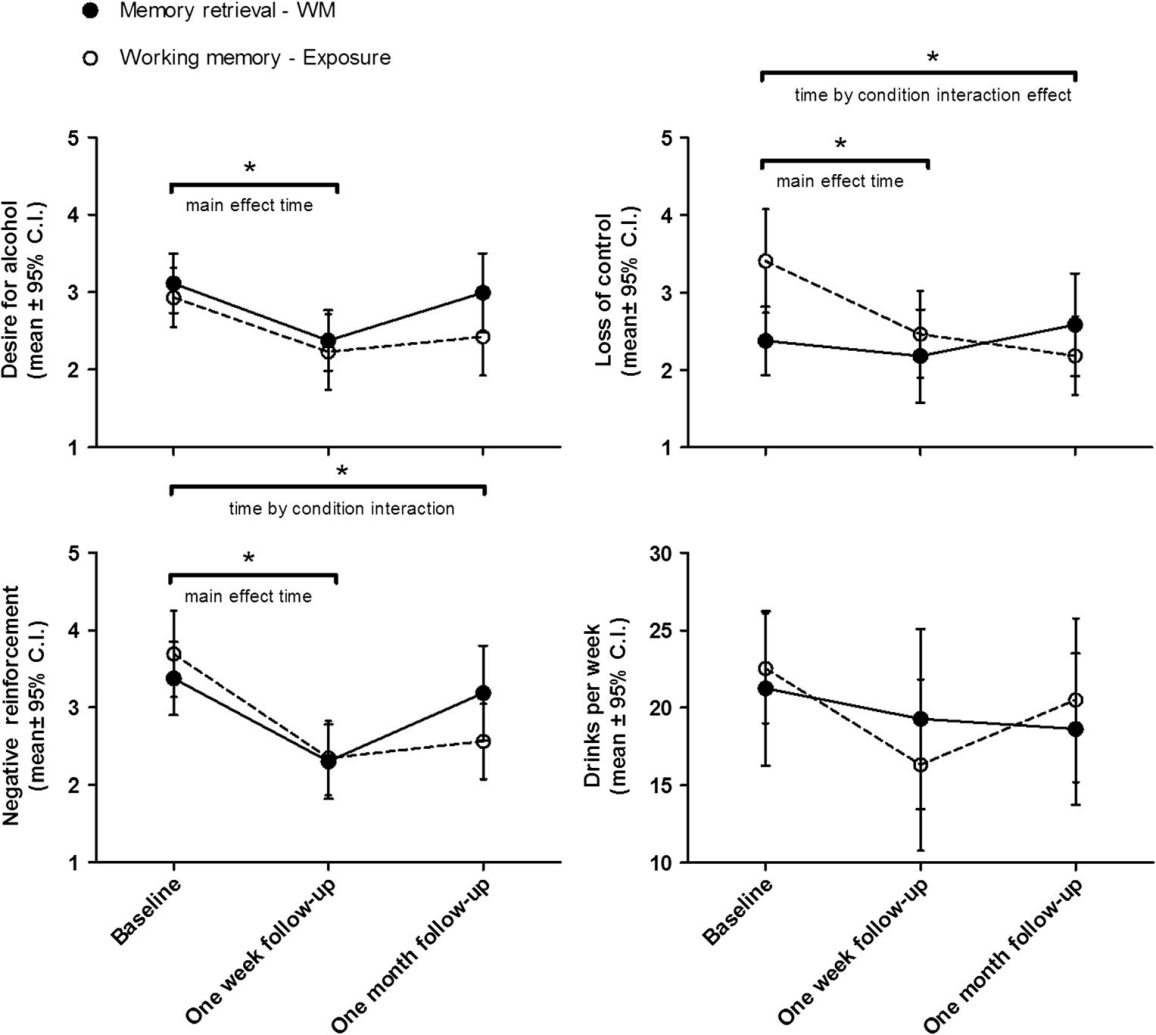
In both groups, there was a significant reduction in desire for alcohol at 1-week follow-up, but this effect did not last until 4 weeks of follow-up. However, there was a significant time by condition interaction effect for DAQ-control and DAQ-negative reinforcement, as only participants in the WM-memory retrieval condition displayed a reported a significant reduction in craving related to negative reinforcement and loss of control at 1-month after the intervention. No significant reductions in alcohol intake were reported

Repeated measures ANOVAs for the three different subscales demonstrated that the condition by time interaction effect was mainly driven by changes in craving related to negative reinforcement and craving related to loss of control (Fig. 6). For DAQ-negative reinforcement, there was a significant main effect of time (*F*_2, 104_ = 20.39, *p* < 0.001, *η*_p_^2^ = 0.28) and a significant time by condition interaction effect (*F*_2, 104_ = 3.39, *p* = 0.04) (Fig. 5. Planned comparisons revealed that there was a significant reduction from baseline in DAQ-negative reinforcement at 1-week follow-up (*F*_1, 52_ = 35.48, *p* < 0.001, *η*_p_^2^ = 0.41) and 1-month follow-up (*F*_1, 52_ = 10.26, *p* = 0.002, *η*_p_^2^ = 0.17). Also, there was a significant time by condition interaction effect at 1-month follow-up compared to baseline (*F*_1, 52_ = 5.30, *p* = 0.025, *η*_p_^2^ = 0.10) as the WM-MR group showed a significant reduction from in DAQ-negative reinforcement at 1-month follow-up compared to baseline (*F*_1, 25_ = 17.71, *p* < 0.001, *η*_p_^2^ = 0.42), whereas the MR-WM group did not.

For DAQ-control, there also was a main effect of time (*F*_2, 104_ = 11.78, *p* < 0.001, *η*_p_^2^ = 0.19) and a significant time by condition interaction effect (*F*_2, 104_ = 6.44, *p* = 0.002, *η*_p_^2^ = 0.11). Planned comparisons showed that there was a significant reduction in DAQ- (loss in) control at 1-week follow-up compared to baseline (*F*_1, 52_ = 9.88, *p* = 0.003, *η*_p_^2^ = 0.16) and at 1-month follow-up compared to baseline (*F*_1, 52_ = 18.78, *p* < 0.001, *η*_p_^2^ = 0.27). In addition, there was a time by condition interaction effect at 1-month follow-up compared to baseline (*F*_1, 52_ = 10.56, *p* = 0.002, *η*_p_^2^ = 0.17). Within group analyses revealed that only in the WM-MR group there was a significant reduction in DAQ-control at 1-month follow-up compared to baseline (*F*_1, 25_ = 25, *p* < 0.001, *η*_p_^2^ = 0.50).

For DAQ-desire, there was a main effect of time (*F*_2, 104_ = 7.81, *p* = 0.001, *η*_p_^2^ = 0.13) but no main effect of condition or a time by condition interaction effect. Planned comparisons showed that there was a significant reduction in DAQ-desire at 1-week follow-up in comparison with baseline (*F*_1, 52_ = 19.59, *p* < 0.001, *η*_p_^2^ = 0.27) but not at 1-month follow-up.

For weekly alcohol intake, there were no significant main or interaction effects. However, planned comparisons demonstrated a significant reduction from baseline at 1-week follow-up (*F*_1, 50_ = 5.49, *p* = 0.023, *η*_p_^2^ = 0.10) but not at 1-month follow-up (Fig. 6). Altogether, these results suggest that a high WM load preceding three alcohol memory retrieval session has a long-term effect on alcohol craving related to negative reinforcement and loss of control but not on weekly alcohol intake.

### The direct effect of a high WM load on retrieval-induced craving, heart rate, skin conductance levels, and vividness of the retrieved memories

#### Differences between conditions in retrieval-induced desire for alcohol during the three intervention sessions

Repeated measures ANOVA on the total DAQ scores demonstrated a significant retrieval-induced increase in total DAQ scores over all three training sessions (*F*_1, 52_ = 44.93, *p* < 0.001, *η*_p_^2^ = 0.46), whereas total DAQ scores significantly declined over sessions (*F*_2, 104_ = 3.92, *p* = 0.023, *η*_p_^2^ = 0.07). Moreover, there was a significant retrieval by session interaction effect (*F*_2, 104_ = 3.20, *p* = 0.045, *η*_p_^2^ = 0.06).

During all three intervention sessions, there was a significant retrieval-induced increase in DAQ-desire (*F*_1, 54_ = 71.69, *p* < 0.001, *η*_p_^2^ = 0.57). Moreover, there was a significant retrieval by session interaction effect (*F*_2, 53_ = 4.11, *p* = 0.022, *η*_p_^2^ = 0.13), as retrieval-induced desire for alcohol reduced over sessions. The other main and interaction effects were not significant. Also for DAQ-reinforcement, there was a main effect of retrieval (*F*_1, 54_ = 12.70, *p* = 0.001, *η*_p_^2^ = 0.19) and session as the overall scores on DAQ-reinforcement gradually reduced over sessions (*F*_2, 53_ = 6.30, *p* = 0.004, *η*_p_^2^ = 0.19). All the other main or interaction effects were non-significant. For DAQ-control, there was only a significant main effect of memory retrieval (*F*_1, 54_ = 8.34, *p* = 0.006, *η*_p_^2^ = 0.13), but the other main or interaction effects were non-significant. Altogether, these results (Fig. S1) demonstrate that there were no differences between intervention conditions in retrieval-induced desire for alcohol during training, suggesting that there is no direct effect of a high WM load on retrieval-induced desire for alcohol. However, the decrease in retrieval-induced desire could indicate a general effect of the number of retrieval sessions on extinction of alcohol-related memories.

#### Differences between conditions in retrieval-induced physiological reactivity during the three intervention sessions

For skin conductance level (Fig. S2), there was significant cubic relation between retrieval phase and skin conductance level (*F*_1, 32_ = 29.38, *p* < 0.001, *η*_p_^2^ = 0.48) and a significant retrieval by condition linear interaction effect (*F*_1, 32_ = 14.56, *p* < 0.001, *η*_p_^2^ = 0.31). Follow-up analyses within each intervention condition revealed that the linear relation between retrieval phase and skin conductance level was stronger in the memory retrieval-WM group (*p* < 0.001, *η*_p_^2^ = 0.69) than in the WM-memory retrieval group (*p* = 0.18, *η*_p_^2^ = 0.36), suggesting that skin conductance levels more strongly increased in the memory retrieval-WM condition, compared to the WM-memory retrieval condition. In addition, there was a significant effect of session (*F*_5, 31_ = 4.59, *p* = 0.018, *η*_p_^2^ = 0.23) and a significant session by condition interaction effect (*F*_2, 31_ = 4.00, *p* = 0.28, *η*_p_^2^ = 0.21). Post hoc follow-up tests demonstrated that there was a main effect of session in the WM-memory retrieval group only (*F*_2, 12_ = 5.29, *p* = 0.022, *η*_p_^2^ = 0.47). The other main or interaction effects were non-significant (see Fig. S2).

For heart rate (Fig. S2), there was also a significant quadratic effect of retrieval phase (*F*_1, 31_ = 9.33, *p* = 0.005, *η*_p_^2^ = 0.23) and a significant session by condition interaction effect (*F*_2, 30_ = 3.6, *p* = 0.04, *η*_p_^2^ = 0.19). Post hoc analyses demonstrated that there was a significant effect of session only in the MR-WM group (*F*_2, 18_ = 4.06, *p* = 0.35, *η*_p_^2^ = 0.11) (see Fig. S2).

#### Differences between conditions in working memory capacity during the three intervention sessions

Overall, the mean length of the chessboard sequence was 7.2 (e.g., a high WM load of 7.2 items to keep online). Repeated measures analyses demonstrated that there was a significant increase in the length of the chessboard sequence during the task (*F*_3, 132_ = 6.70, *p* = 0.001, *η*_p_^2^ = 0.12) as well as over sessions (*F*_2, 83_ = 10.5, *p* < 0.001, *η*_p_^2^ = 0.17). Moreover, there was a session by phase interaction effect (*F*_5, 300_ = 3.10, *p* = 0.009, *η*_p_^2^ = 0.06). Follow-up analyses revealed that there was a significant increase in sequence length during the training only in the first training session (*F*_3, 153_ = 11.36, *p* < 0.001, *η*_p_^2^ = 0.18) (Fig. S3). Importantly there were no session by condition, phase by condition, or session by phase by condition interaction effects, demonstrating that both intervention groups performed equally well on the WM training (Fig. S3).

The number of sequence errors made significantly increased during the task (*F*_2, 100_ = 8.05, *p* = 0.001, *η*_p_^2^ = 0.14) but decreased over training sessions (*F*_3, 150_ = 83.24, *p* < 0.001, *η*_p_^2^ = 0.63). In addition, the number of rectangle errors made during the WM task significantly reduced over training sessions (*F*_2, 100_ = 5.8, *p* = 0.004, *η*_p_^2^ = 0.10). The other main and interaction effects were non-significant, demonstrating again that there were no differences in WM capacity between intervention conditions.

### Differences between conditions in vividness and the capacity to recall the memories

When comparing sessions 1 and 5, there was a significant condition by session interaction effect (*F*_1, 55_ = 5.29, *p* = 0.025, *η*_p_^2^ = 0.09) on the vividness of the memory, as there was a significant reduction in the vividness of the memory in the WM-MR condition (*F*_1, 26_ = 12.28, *p* = 0.002, *η*_p_^2^ = 0.32) but not in the MR-WM condition (*F*_1, 29_ = 2.58, *p* = 0.12, *η*_p_^2^ = 0.08). The capacity to imagine themselves into the situation significantly reduced in both conditions (*F*_1, 55_ = 22.53, *p* < 0.001, *η*_p_^2^ = 0.29).

There were no differences between intervention conditions in retrieval-induced desire for alcohol during training. However, while participants in the MR-WM groups showed a strong increase in SCL during memory retrieval, this effect was not present in the WM-MR group (significant interaction effect), suggesting less retrieval-induced arousal in the WM-MR group. No such interaction effect was shown for retrieval-induced changes in heart rate.

### Exploratory analyses: the relation between WM load and changes in retrieval-induced craving and craving at follow-up

To explore the relation between WM task performance and changes in retrieval-induced responses and craving at follow-up, all participants were categorized to a low or high WM load condition, based on their WM performance. In the low WM load group, the length sequence ranged from 4.64 to 7.28, and in the high WM load group, the length sequence ranged from 7.35 to 9.31. WM load was subsequently added to the previous analyses.

Exploratory analyses demonstrated that there was a significant interaction between retrieval, session, and WM load on DAQ-total scores (*F*_1, 52_ = 8.60, *p* = 0.005, *η*_p_^2^ = 0.14) but not on the three different subscales. Follow-up analyses demonstrated that there was a significant retrieval by session interaction effect only for the participants with the highest WM load (*F*_1, 25_ = 22.93, *p* < 0.001, *η*_p_^2^ = 0.55), as there was a significant effect of retrieval on craving only in session 1 (*F*_1, 26_ = 39.99, p < 0.001, *η*_p_^2^ = 0.61) and not in session 5. These results suggest that individuals with a high WM capacity may benefit more from a WM intervention or cue-exposure intervention, independent of the order of these interventions.

Exploratory analyses on craving at follow-up demonstrated no significant interaction with WM capacity or retrieval-induced changes in heart rate, skin conductance, and vividness of the retrieved memories.

## Discussion

In this study, we investigated the effectiveness of a novel 3-day MR-WM intervention in a population of non-treatment seeking problem drinkers. It was hypothesized that a high WM load following alcohol-related memory retrieval would disrupt the reconsolidation of those memories, leading to reductions in retrieval-induced subjective craving and physiological reactivity as well as reductions in craving and alcohol intake 4 weeks later. However, we did not find any experimental support for this. In contrast, and perhaps surprisingly, we did find that a high WM load *prior* to memory retrieval (the original control condition) significantly reduced retrieval-induced desire for alcohol, as well as alcohol craving related to negative reinforcement and behavioral control at 1-month follow-up. However, we did not observe a significant reduction in alcohol intake. Also, retrieval-induced SCL were most strongly modified in the MR-WM condition. During the intervention sessions, we observed that a high WM load prior to memory retrieval led to a smaller increase in SCL during retrieval, which could be related to less emotional arousal induced by memory retrieval.

The findings of the current study do not support our original hypothesis that a high WM load interferes with the reconsolidation of alcohol-related memories. While the WM task took place immediately after memory retrieval and within the reconsolidation window, it is difficult to assess on a behavioral level whether reconsolidation actually took place (Hutton-Bedbrook and McNally 2013). Previous studies suggest that the critical condition for inducing memory reconsolidation, instead of memory extinction, is a prediction error (e.g., a mismatch between what is expected based on previous experiences and the actual state of events) (Sevenster et al. 2014). It has previously been demonstrated that an instruction “not to drink” after participants have been instructed to prepare a drink successfully induces a prediction error in hazardous drinkers (Das et al. 2015b). Similarly, we aimed to induce a prediction error by instructing the participants to drink the alcohol in session one, while they did not receive this instruction during the following three intervention sessions. In fear learning, it has, however, been found that *too much* new learning due to multiple prediction errors actually no longer triggers memory reconsolidation (Sevenster et al. 2014). Moreover, it could be argued that in our current design, participants in sessions 3 and 4 were already aware of the fact that they would not receive an instruction to drink alcohol, which would have diminished the prediction error. Hence, reconsolidation may have taken place only in the second session, while in the remaining intervention sessions, a process of extinction was induced. As we observed a significant reduction in (retrieval-induced) craving in the WM-MR condition only, this could imply that a high WM load prior to memory retrieval enhances extinction learning.

Indeed, recent findings suggest that enhanced extinction learning may be more resistant to various forms of drug reinstatement compared to normal extinction (Janak et al. 2011; Kearns et al. 2012; Millan et al. 2013). In these studies, enhanced extinction learning was achieved by either noradrenergic modulation (Janak et al. 2011) or through the presentation of already extinguished cues during extinction learning (Rescorla 2006; Kearns et al. 2012). Therefore, a high WM load *prior* to memory retrieval may have indeed induced enhanced extinction, resulting in reduced (retrieval-induced) craving for alcohol that lasts up to 4 weeks after the intervention. The retrieval-induced changes in SCL during the three intervention sessions provide further evidence for this as retrieval-induced SCL in the WM-MR condition was significantly smaller compared to the MR-WM condition. Furthermore, while SCL in the WM-MR condition returned to baseline levels near the end of the retrieval sessions, they remained high in the MR-WM condition. Although enhanced extinction was not observed in self-reported desire for alcohol, the SLC data could be indicative of enhanced extinction of the conditioned responses in the WM-MR condition but not in the MR-WM condition.

Alternatively, the current findings could be explained by the WM theory proposed by Andrade et al. (1997) stating that WM capacity is required to recall memories. As a consequence, if other resources of WM are utilized during memory reactivation, the vividness and emotionality of these memories are reduced, which will result in these memories to be updated into a less emotional form (Nader 2007; Schwabe et al. 2014; Littel et al. 2016). Indeed, it has been observed that a high (visual spatial) WM load during the retrieval of substance-related memories reduces craving for cigarettes (May et al. 2010) and food (McClelland et al. 2006; Steel et al. 2006; Andrade et al. 2012; Kemps and Tiggemann 2013). In a clinical equivalent of these studies, an Eye Movement Desensitization and Reprocessing (EMDR) procedure has been used, showing immediate reductions in cigarette cravings (Markus et al. 2016). Similar effects of EMDR have been found on food cravings (Littel et al. 2016). While a reduction in craving in these studies is often interpreted to be a results of impaired memory reconsolidation (Markus et al. 2016), it has been suggested that a high WM load only effectively reduces craving when it *prevents* the activation of the (craving-inducing) memories (Van Dillen et al. 2013). In other words, a high WM load should be induced prior to memory retrieval, as was the case in the WM-MR condition, instead of after memory reactivation, as was the case in the MR-WM condition, in order to reduce the emotionality of the memories.

It should be noted though that in the current study, the WM task took place *prior* to memory retrieval and not *during* memory retrieval. Therefore, the WM task could only have competed with memory retrieval, and thus emotionality of the retrieved memories, if the high WM load lasted throughout the memory retrieval session. However, there is currently no empirical evidence for such a lasting effect of a WM task on brain activity in regions involved in memory retrieval, including the prefrontal cortex. Hence, the conclusion that a high WM load prior to memory retrieval reduces the emotionality of the retrieved memories should be taken with caution. Nonetheless, we did observe smaller SCL responses during memory retrieval in participants who performed a WM task *prior* to alcohol-related memory retrieval, compared to participants who performed a WM task immediately *after* memory retrieval. An increase in SCL has previously been reported in response to addiction-related cues in smokers (Field and Duka 2004; Bailey et al. 2010; Cortese and Uhde 2016), patients with an eating disorder (Gorini et al. 2010), cannabis users (Norberg et al. 2016), and opiate addicts (de Quiros Aragon et al. 2005) and is generally seen as an index of objective measure of emotional arousal (Connelly and Denney 2007; Lang et al. 1993). These findings therefore support the hypothesis that a high WM load *prior* alcohol-related memory retrieval reduces the emotionality of the memories. Importantly, it has been suggested that a high WM during memory retrieval results in a blurred version of the original memory to be reconsolidated (van den Hout et al. 2014), which explains the (long-lasting) reduction in self-reported craving of participants in the WM-MR condition.

Changes in self-reported craving, SCL, and heart rate during the assessment sessions were somewhat counterintuitive. That is, retrieval-induced SCL during sessions 1 and 5 was only significantly changed in the MR-WM condition, while significant reductions in craving are only observed in the WM-MR condition. However, conditions already differed in retrieval-induced SCL at baseline, making the interpretation of this interaction effect difficult. Moreover, there was no intervention effect on retrieval-induced changes in heart rate, and heart rate was increased as decreased during different phases of retrieval. Previous studies also failed to report effects of memory retrieval (cue-exposure) on heart rate in smokers (Bailey et al. 2010; Cortese and Uhde 2016). Therefore, heart rate may not be the most sensitive measure of cue-reactivity.

This study has several limitations. First of all, to rule out all possible alternative explanations for our results, additional control groups are needed. For example, whereas we expected that a high WM load during memory reconsolidation would impair the reconsolidation of alcohol-related memories, a high WM load could also have strengthened the reconsolidation of alcohol memories, preventing a spontaneous reduction in craving as it could have occurred over time. Indeed, such unexpected timing effects have previously been demonstrated using pharmacological reconsolidation blockers (Torregrossa and Taylor 2016). Furthermore, if reconsolidation actually took place, the internal state (arousal induced by the WM-task) could have been incorporated into the labialized memory trace (context-dependent memory) such that it is only accessible in such a state. These effects have previously been demonstrated with pharmacological manipulations (Gisquet-Verrier et al. 2015). Moreover, based on the current study design, we cannot exclude the possibility that simply doing a task prior to memory retrieval, instead of specifically taxing the WM, affects cue-induced craving. Hence, future studies should be conducted to exclude these alternative explanations, such as include an MR-only condition, a WM-only condition, a non-WM related task + MR condition, and/or a WM task prior to testing, to exclude these possibilities. Secondly, as non-treatment seeking problem drinkers were included, it should be investigated how the current findings translate to a clinical population. In addition, the WM-MR condition reported slightly higher use of cannabis and MDMA. However, there were no baseline differences on other, more relevant, measures, such as alcohol intake, alcohol use severity, retrieval-induced craving, or WM capacity. Moreover, there is no reason to assume that a WM-MR intervention aimed at reducing alcohol craving and consumption is more effective in cannabis/NDMA users. Therefore, these baseline differences are unlikely to have biased our findings.

To our knowledge, this is the first study to investigate the potential of a behavioral intervention at disrupting the reconsolidation of addiction-related memories. Whereas WM interference on drug exposure has been investigated before, these studies used dual WM-exposure tasks, making the interpretation of the findings difficult (Andrade et al. 2012; Kemps and Tiggemann 2013; May et al. 2010; McClelland et al. 2006; Steel et al. 2006). That is, in these studies, reductions in craving could have been the result of impaired memory activation or the disruption of the memory reconsolidation process (Nader 2007; Schwabe et al. 2014; Littel et al. 2016). In the current study, however, participants performed a WM task prior or immediately following the memory retrieval session, demonstrating that reductions in craving due to WM-MR interference are likely to be due to impaired memory activation rather than the disruption of memory reconsolidation, although reconsolidation could also have been affected in this latter condition (van den Hout et al. 2014). Moreover, in contrast to most previous studies, we included three (instead of one) intervention sessions, as well as follow-up measures of craving and alcohol intake which provide a more complete view on the long-term effects of WM-MR interventions. Another strength is that both males and females were included in the study and that these results are therefore easily translatable to both genders.

In summary, the aim of this study was to investigate the potential of a WM load to interfere with the reconsolidation of alcohol-related memories. In contrast to our expectations, a WM task following memory retrieval did not interfere with the reconsolidation of alcohol-related memories. We did find that a high WM load *prior* to memory retrieval significantly reduces (retrieval-induced) craving for alcohol that lasted up to 4 weeks during follow-up. These reductions are likely to be due to WM competition during memory activation, reducing the vividness and emotionality of the memories by which they are updated to a less emotional form (Nader 2007; Schwabe et al. 2014; Littel et al. 2016). It remains to be investigated whether WM-induced changes in the prefrontal cortex last indeed long enough to interfere with the retrieval of alcohol-related memories 15 min later and which exact mechanism (extinction, reconsolidation, other?) underlies the change in the original maladaptive memories. Therefore, future studies are needed to better understand the neural mechanisms behind the effectiveness of the WM-MR intervention, as it may have potential to become an effective (adjunctive) intervention in the treatment of SUD.

## Electronic supplementary material

ESM 1 (DOCX 217 kb)
