# Supplementary material for: A high working memory load prior to memory retrieval reduces craving in non-treatment seeking problem drinkers
Source: Psychopharmacology (Berl). 2017 Nov 27;235(3):695–708. doi: 10.1007/s00213-017-4785-4 (PMC5847068; doi:10.1007/s00213-017-4785-4)
Supplement: Supplementary file 1 — (DOCX 217 kb) [file 213_2017_4785_MOESM1_ESM.docx]

A high working memory load prior to memory retrieval reduces craving in non-treatment seeking problem drinkers Anne Marije Kaag, PhD^12^, Professor Anna E Goudriaan^3^_,_ Professor Taco J de Vries^4^, Tommy Pattij, PhD^4^, Professor Reinout W. Wiers^12^

^1^Addiction Development and Psychopathology (ADAPT) lab, department of psychology, University of Amsterdam, the Netherlands

^2^Amsterdam Brain and Cognition, University of Amsterdam

^3^Departement of Psychiatry, Academic Medical Center, Amsterdam, the Netherlands

^4^Department of Anatomy and Neurosciences, Amsterdam Neuroscience, VU University Medical Center, Amsterdam, The Netherlands

**Corresponding author:**

Anne Marije Kaag

Tel: +31 61 81 60 587

[amkaag@gmail.com](mailto:amkaag@gmail.com)

# Supplementary Methods

1. **Memory retrieval task**

The computer-assisted alcohol memory retrieval paradigm was modified from a protocol by Hammarberg and colleagues (Hammarberg, Jayaram-Lindström, Beck, Franck, & Reid, 2009; Khemiri et al., 2015). The memory retrieval session started with the presentation of the 14 items of the DAQ, that were scored on a 7-point Likert scale. Thereafter, for 75 seconds, participants underwent a voice-guided relaxation exercise that was presented through headphones to get a baseline measure of skin conductance levels and heartrate. Next, the actual memory retrieval started with the presentation of four alcohol-related pictures, that were all shown for 30 seconds, with an inter-stimulus interval of 4-6 seconds. Two of these stimuli were from the Amsterdam Beverage Picture set (Pronk, van Deursen, Beraha, Larsen, & Wiers, 2015), and either contained a picture of beer, a picture of white wine or a picture of red wine, depending on the preference of the participant. The other two pictures were personal pictures from the participant, of situations that were strongly craving inducing, and also displayed an alcoholic beverage. These pictures were made by the participants themselves and sent to the lab prior to the intervention. Subsequently, participants were shown two personalized scenarios of alcohol-related situations. These scenarios were based on information provided by the participants prior to the start of the experiment. The first scenario referred to a pleasant alcohol-related memory and the second scenario referred to a situation that induced a strong feeling of craving. Participants were instructed to read these scenarios out loud and to imagine themselves in the situation. The scenarios (with an approximate length of 70 words) were presented for 30 seconds, with an inter-stimulus interval of 4-6 seconds. This imagery phase was followed by an in-vivo exposure phase. This phase started with the instruction to open a box that was in front of them, and to take out the alcoholic beverage and glass (beer or wine, depending on the preference of the participant). Thereafter participants were instructed to pour the drink into the glass and to bring the glass to their nose and to smell the beverage, for three times. Finally, the participants were instructed to take a sip of the alcohol. Participants had 30 seconds to perform each instruction (take the alcohol, poor the alcohol, smell the alcohol and drink the alcohol). At the end of the memory retrieval sessions the 14 items of the DAQ were shown on the computer screen and participants had to rate their craving on the 7-point Likert scale. Additionally, participants had to rate the vividness and the capacity to imagine themselves in the scenarios on a 10-point Likert scale.

It has been suggested that a difference between predicted outcomes and the actual experienced outcomes (that is, a prediction error) is critical to destabilize memories and to induce memory reconsolidation (Fernandez, Boccia, & Pedreira, 2016; Sevenster, Beckers, & Kindt, 2014; Taylor & Torregrossa, 2015). Therefore, the memory retrieval protocol in session two, three and four did not include the last instruction to drink the alcohol (compliance to this instruction was confirmed by observing the participants). Doing so we aimed to manipulate a prediction error as participants were anticipating on drinking the alcohol (as they did in the first session), whereas in fact the protocol stopped after the instruction to smell the alcoholic beverage.

**Complex chessboard task**

The complex chessboard task, developed by Dovis, Van Der Oord, Wiers, & Prins ( 2012), is a visuo-spatial working memory task based on the Corsi block tapping task (Corsi, 1973) and the subtest Letter-Number Sequencing from the Wechsler Adult Intelligence Scale (WAIS; Wechsler, 1958). The task, described in figure 3, assesses the ability to both maintain and manipulate/reorganize visual-spatial information that is relevant for the task at hand. The complex chessboard task starts with the stimulus presentation of a rectangle trial. Participants are instructed to remember the sequence of the rectangles lighting up. Subsequently the colored grid appears and a sequence of squares light up one by one. Each square lights up for 900ms and is followed by an inter-stimulus interval of 500ms. After this sequence is shown, the participant has to repeat the sequence by mouse-clicking on the squares in a reorganized way: The green stimuli have to be reproduced before the blue stimuli; both in the same order as presented. After the response, feedback is presented: positive feedback consisted of the presentation of a €10 bill, a green checkmark and the text “more chance at winning €10” and negative feedback consisted of the presentation of a €10 bill, a red cross and the text: “less chance at winning €10”. The participants were told that if they were shown a green screen after completion of the task they would receive an additional €10 after study completion. Two other chessboard trials followed thereafter. After the third chessboard trial, the grid turns grey and the rectangles reappear; the participant should press the green button before he/she reproduces the rectangles (this is the only moment that pressing the green button is correct). Next, the participant has to reproduce the stimulus presentation of the rectangles trial in the same order as presented. After the response, feedback is given and the task continues to the next block. The difficulty level of the task was adaptive; the first sequence consisted of two stimuli and after two consecutive correct reproductions, the sequence was increased by one stimulus. After two consecutive incorrect reproductions, the sequence was shortened by one stimulus. As a consequence all participants would see a green screen after completion of the task and receive €10, to keep all participants motivated. The minimum sequence length consisted of two stimuli and there was no maximum sequence length. Participants performed a total of 76 trials (19 rectangle trials and 57 chessboard trials) that took approximately 30 minutes.

### Skin conductance and heart rate measurements

ECG was measured using a custom made portable amplifier with a 1GΩ input resistance and a bandwidth of 0.1Hz (6dB/oct) to 250Hz (24 dB/oct) containing a National Instruments NI-USB6210 A/D converter to digitize the analogue data at a rate of 1000 S/s. Vsrrp98 software was used to record and analyze the data (Vsrrp98 v10.4, University of Amsterdam, 1998-2017). We used disposable pregelled Ag/AgCl 3M Red Dot electrodes that were placed according to the Einthoven triangle LEAD-II method (Abi-Saleh & Omar, 2010) in which one lead is placed just below the left collarbone, the ground is placed just below the right collarbone, and the second lead is placed on the lower right side of the chest. The electrodes for the skin-conductance measurement were placed on the tip of the left index- and middle finger. Vsrrp98 detects r-tops from the ECG recording and calculates heart rate. SCL was measured with a portable amplifier by means of a sine wave shaped excitation voltage (1V pk-pk, 50Hz) on the electrodes. Electrodes were custom made curved Ag/AgCl electrodes with a dimension of 20x16mm. Skin conductance and hear rate were measured continuously during the retrieval sessions.

# Supplementary Results (figures)

**Figure S1.** For all three subscales of the DAQ there was a main effect of retrieval. In addition, retrieval-induced craving related to desire and negative reinforcement gradually reduced over the three training sessions. Importantly there were no significant differences between treatment conditions.


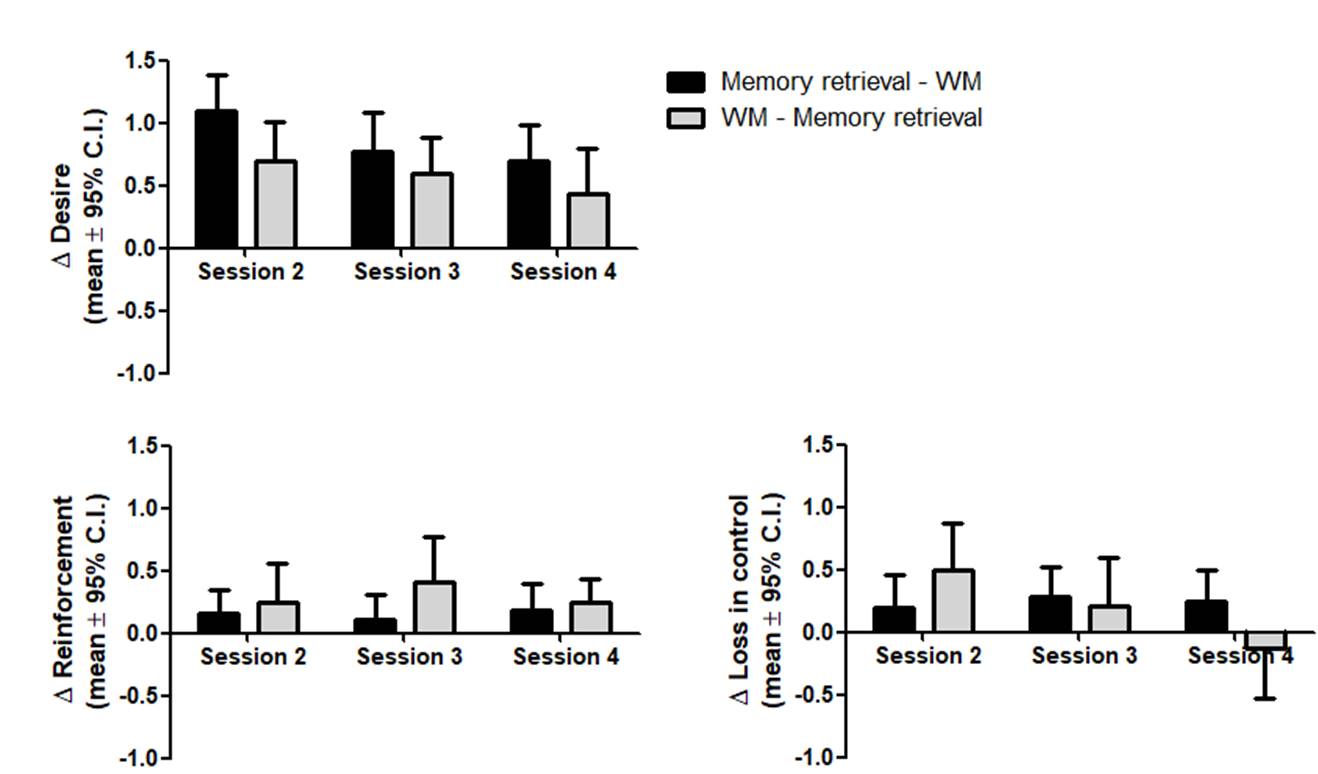


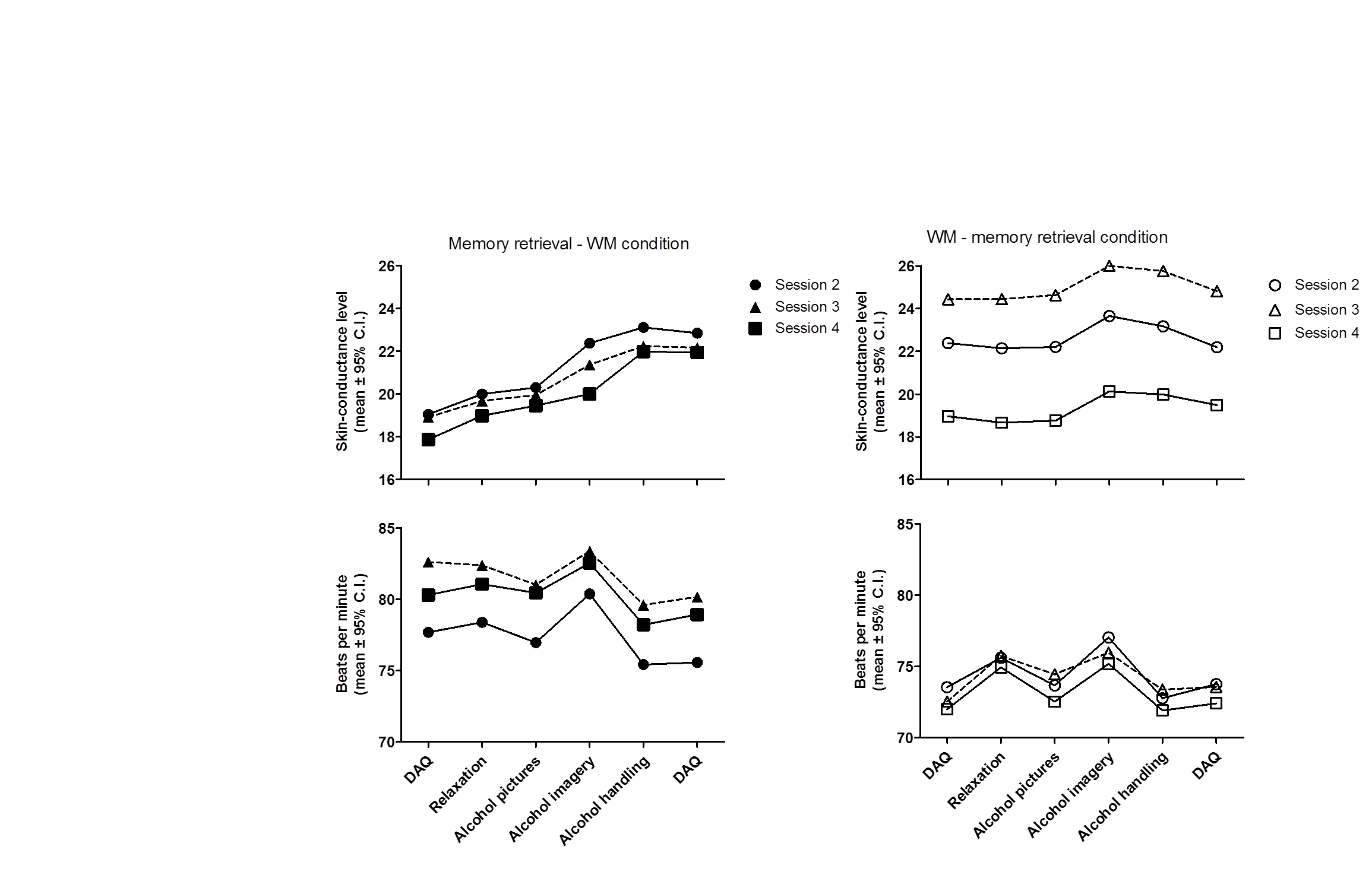


**Figure S2.** During all three intervention sessions, skin conductance levels increased linearly during memory retrieval, only in the memory retrieval –WM condition. In the WM-memory retrieval condition skin conductance levels increased only slightly, quickly returning back to baseline levels after memory retrieval. For heart rate, there was a main effect of retrieval and a significant session by condition interaction effect. That is, only in the memory retrieval-WM condition there was a main effect of session, but this was irrespective of memory retrieval.


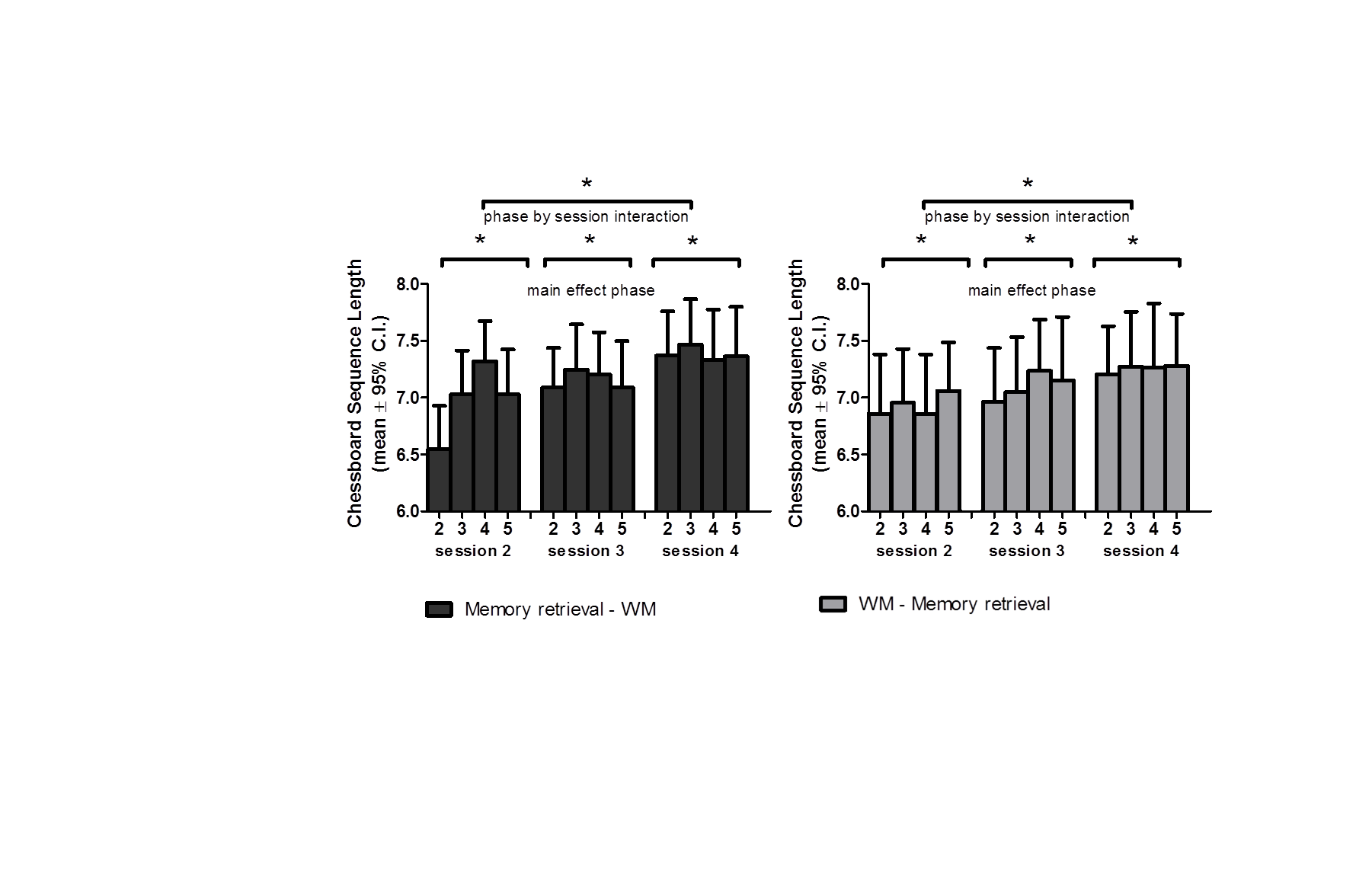


**Figure S3.** For both conditions, the sequence length of the chessboard trials increased during the four phases of the WM task, indicative of increased WM capacity. Moreover, there was a phase by session interaction effect, as the sequence lengths of the chessboard trials increased with each session. There were no significant differences between condition, demonstrating that both groups displayed similar (increases in) working memory capacity.
